# Salvage High-Intensity Focused Ultrasound for Prostate Cancer after Radiation Failure: A Narrative Review

**DOI:** 10.3390/curroncol31070270

**Published:** 2024-06-26

**Authors:** Sina Sobhani, Anosh Dadabhoy, Alireza Ghoreifi, Amir H. Lebastchi

**Affiliations:** Institute of Urology, Norris Comprehensive Cancer Center, University of Southern California, Los Angeles, CA 90089, USA; ssobhani@usc.edu (S.S.); anosh.dadabhoy2@med.usc.edu (A.D.); alireza.ghoreifi@med.usc.edu (A.G.)

**Keywords:** (MeSH) salvage therapy, high-intensity focused ultrasound, prostate cancer, cancer recurrence, radiotherapy, minimally invasive surgical procedures

## Abstract

For patients diagnosed with localized prostate cancer, there are multiple treatment options available. The traditional treatment modalities include radical prostatectomy and radiotherapy. Nevertheless, focal therapy, including high-intensity focused ultrasound (HIFU) and cryotherapy, has emerged as a less-invasive method in this setting. Some patients undergoing primary radiation therapy experience recurrence, but there is currently no consensus on the optimal approach for salvage treatment in such cases. The lack of robust data and randomized controlled trials comparing different whole-gland and focal salvage therapies presents a challenge in determining the ideal treatment strategy. This narrative review examines the prospective and retrospective data available on salvage HIFU following radiation therapy. Based on the literature, salvage HIFU for radio-recurrent prostate cancer has promising oncological outcomes, with an overall 5-year survival rate of around 85%, as well as incontinence rates of about 30% based on the patient’s risk group, follow-up times, definitions used, and other aspects of the study. Salvage HIFU for prostate cancer proves to be an effective treatment modality for select patients with biochemical recurrence following radiotherapy.

## 1. Introduction

Prostate cancer (PCa) is the second leading cause of cancer mortality for men in the United States, with about 288,000 new cases and 34,500 deaths projected for 2023. It is the most commonly diagnosed cancer in men after dermatological malignancies, and will affect one in every eight men over their lifetime [1]. Given the growing incidence and the relatively substantial survivorship among PCa patients, the burden of this disease increasingly contributes to our population’s economic burden [1,2,3]. The role of effective screening, early detection, and management is crucial in the detection of PCa; however, complete consensus surrounding screening guidelines does not exist among different providers and institutions [4,5]. When weighing the benefits of early detection against the risk of over-diagnosis, these guidelines are critical in providing a framework for informed decision-making.

It has been reported that more than 70% of PCa cases are diagnosed at a localized stage, with a five-year survival rate approaching 100% [1]. The National Comprehensive Cancer Network (NCCN) guidelines for PCa lay out various treatment strategies tailored to different risk groups, stratified based on serum PSA, digital rectal exam, imaging, and biopsy [6]. These treatment options include active surveillance, external beam radiotherapy (EBRT), androgen deprivation therapy (ADT), radiation therapy (RT), brachytherapy (BT), and radical prostatectomy (RP) [7]. The main treatment options for patients with clinically localized PCa are RP and radiotherapy. Both modalities are associated with acceptable oncological outcomes, yet with significant treatment-related side-effects [8,9]. Focal therapy (FT) has emerged as an alternative option, with the aim of improving quality of life by reducing side-effects without compromising cancer control [10,11]. When choosing between focal therapy and whole-organ treatment, the patient’s disease risk group and post-treatment potential quality-of-life risks are important factors that guide shared decision-making between the patient and their physicians. A significant number of patients ultimately end up selecting FT as an alternative to radical treatments, given their adverse events and associated complications [12,13].

Although different treatment modalities provide acceptable cancer-free survival and oncological outcomes, a significant subset of patients still experience recurrence. After primary treatment of localized PCa with radiation, about a third of patients (depending on radiotherapy dose) have been reported to show rising PSA levels within 5 years without clinical evidence of disease (i.e., biochemical recurrence: BCR) [14]. The American Society for Therapeutic Radiology and Oncology (ASTRO) defines BCR after external beam radiation therapy (EBRT) as the occurrence of three consecutive PSA rises after a nadir, while the Phoenix definition considers BCR as a serum PSA increase of 2 ng/mL or greater above the nadir level [15].

BCR after primary radiotherapy poses a complex challenge, and there is no unanimously accepted standard of care for locally recurrent PCa after failed radiotherapy [16]. Some consider salvage RP as the definitive treatment for patients with radio-resistant PCa; however, this procedure is uncommonly performed given its high morbidity rate, risk of bowel injury, and overall concern for safety [17,18]. Alternative options include reirradiation, salvage high-intensity focused ultrasound (S-HIFU), and cryoablation [16,19,20,21]. Previously explored in relation to various malignancies including brain and breast tumors, HIFU is a treatment modality that utilizes sonic waves to ablate target cells within a tissue using thermal and mechanical energy, with the goal of minimizing damage to surrounding cells. The utilization of salvage therapies is limited due to a scarcity of comprehensive data, a lack of quality data on survival improvement, and concerns over associated adverse effects [22]. It has also been suggested that these therapies might be under-studied or not reported, owing to the burden of adverse consequences and lack of general consensus on a treatment algorithm for this disease. This study aims to explore a sparsely investigated landscape and review the current outlook on the utilization of salvage HIFU for radio-recurrent PCa, as well as the associated oncological and functional outcomes.

## 2. Methods 

We conducted a targeted, non-systematic literature search on PubMed, focusing on contemporary articles related to our topic published in English from 2013 onwards. We found 14 original studies that examined the safety, effectiveness, and oncological and functional outcomes of S-HIFU for radio-recurrent PCa.

Key variables extracted from each study included study type, country of origin, S-HIFU treatment approach (focal or whole-gland), sample size, median age, type of primary radiation treatment, androgen deprivation therapy, and time from primary treatment to salvage. This allowed for a detailed evaluation of patient selection criteria, including disease risk categories and preoperative PSA levels, which are crucial for understanding oncologic outcomes, such as overall and recurrence-free survival rates following S-HIFU.

The collected data were synthesized in order to determine the efficacy and safety of S-HIFU for radio-resistant PCa patients. Perioperative and long-term complications, with a detailed examination of lower tract symptoms, bladder obstruction rates, and rare but significant complications, were also examined, providing a comprehensive view of treatment impact. Functional outcomes were tracked using a variety of methodologies, but overall, the International Prostate Symptom Score (IPSS) and the International Index of Erectile Function (IIEF) questionnaires were used to assess pre- and postoperative urinary and sexual functions, respectively.

## 3. Results and Discussion

A total of 13 studies were included [20,23,24,25,26,27,28,29,30,31,32,33,34], most of which had a relatively small sample size, reflecting the low rate of S-HIFU utilization in the current clinical environment (Table 1). Only two studies contained sample populations in the hundreds, and most of the selected studies used biochemical failure as described by the Phoenix criteria of a PSA > nadir + 2 ng/mL. Each patient population was identified with radio-recurrent PCa after EBRT or brachytherapy (BT), with only a fraction of patients receiving the latter. There was a wide degree of heterogeneity in how the oncological outcomes were reported across the studies, making direct comparison challenging (with less than half of the studies using the D’Amico risk classification criteria).

### 3.1. Criteria for Selecting Patients

Stringent patient selection is paramount in the setting of S-HIFU following radiation failure in order to maximize disease control and functional outcomes while minimizing the risk for futile intervention in patients with metastatic or advanced disease. Although there was some heterogeneity regarding the exact patient selection criteria for S-HIFU, studies recruited patients with histologically proven local recurrence with no evidence of metastasis or suspicious lymph nodes, as screened by radionucleotide bone scan, and abdominal and pelvic CT. In more recent studies, there was an increased utilization of more advanced imaging techniques including multiparametric MRI (mpMRI), as well as choline or prostate-specific membrane antigen (PSMA) PET/CT, in order to enhance the detection of recurrences prior to targeted biopsy and to screen for occult metastasis [23,35,36]. Despite the lack of high-level evidence in this setting, most experts endorse using mpMRI followed by targeted and systematic biopsy before focal therapy; however, the role of PSMA PET, although controversial, has been gaining more acceptance in recent years [23].

Eligibility for S-HIFU includes BCR per Phoenix definition, negative metastatic screening, and localized disease confirmed with imaging. Those with urethral stricture/bladder neck stenosis, rectal wall thickness > 6 mm, excessive prostate volume (>40 mL), or significant prostate calcifications (>1 cm in diameter) should be counselled with caution [20,27]. There is no exact consensus or clear cut-point for the selection criteria in terms staging, grading, and risk-stratification; however, studies generally favor a Gleason score ≤ 7, PSA under 10 ng/mL, and organ-confined disease (T1–2), suggesting that localized and less-aggressive disease is more amenable to local S-HIFU [20,23].

For patients with S-HIFU failure, there is no direct contraindication solely based on prior S-HIFU failure to prevent a patient from redoing S-HIFU. HIFU can be administered more than once, allowing for adaptations in treatment strategy depending on the progression and recurrence of disease; however, meticulous patient selection is, again, paramount [37].

### 3.2. Oncological and Survival Outcomes

Most of the available papers in this setting have small sample sizes and heterogenous cohorts (Table 2). In a 2013 retrospective analysis of 46 patients who underwent S-HIFU following EBRT, Rouvière et al. found that the 2- and 4-year progression-free survival (PFS) rates were 42% and 31%, respectively [34]. When investigating prognostic factors, PSA value at the time of HIFU ablation and the extension of the tumor anterior to the urethral plane on MRI were found to be significantly associated with PFS. In another early series, Baco et al. looked at the outcomes of hemi-gland S-HIFU in patients with unilateral radio-recurrent PCa. They stratified PFS in 48 patients based on D’Amico risk groups and found that overall survival (OS) was 83% at 12 months and 52% at 24 months [31]. The 18-month PFS rate was significantly linked to the Gleason score, with an 82% survival rate for scores ≤ 7 and 34% for scores ≥ 8 (*p* = 0.047). This rate was also significantly associated with pre-HIFU PSA levels, with an 80% survival rate for levels ≤ 4 ng/mL and 49% for levels > 4 ng/mL (*p* = 0.002). They reported no significant difference in the 18-month PFS between patients with a PSA nadir of ≤0.5 ng/mL and those with a nadir of >0.5 ng/mL (72% versus 56%, *p* = 0.3). In the same year, Song et al. reported data on the outcomes of S-HIFU following EBRT. Using the Stuttgart definition (PSA nadir + 1.2 ng/mL) for BCR, they found a BCR-free rate of 53.8%, with a median follow-up time of 44.5 months. BCR was also correlated with a higher pre-EBRT PSA and pre-HIFU PSA, and a short time to nadir [32]. The 5-year rates for OS and cancer-specific survival (CSS) stood at 88% and 94.4%, respectively. Additionally, a nadir PSA of less than 0.5 ng/mL predicted BCR-free survival (*p* = 0.014).

In 2016, Shah et al. retrospectively studied 50 patients who received whole-gland S-HIFU [29]. The 1-year, 3-year, and 5-year PFS rates were reported as 72%, 40%, and 31%, respectively. However, these rates increased to 86%, 47%, and 37% following the exclusion of PSA non-responders, who were defined as patients with postoperative PSA nadirs greater than 0.5 ng/mL. Soon after, Crouzet et al. compiled the most extensive retrospective analysis, with 418 patients across 15 years [20]. The OS, CSS, and metastasis-free survival (MFS) rates at 7 years were about 72%, 82%, and 81%, respectively, while the BCR-free survival rate was calculated at 5 years to be 49% overall, with pre-EBRT risk stratifications of 58%, 51%, and 36% for the low, medium, and high-risk groups, respectively. The BCR-free survival rate was similar to that of Song et al., despite a much larger sample size, lending more validity to the results gathered between studies [32]. In a more recent study, Nair et al. reported a median OS of 17.4 years for S-HIFU versus 12.8 years for no salvage treatment (NST); however, the differences in CSS and OS were not statistically significant, likely due to a reduced sample size and shorter follow-up period in the S-HIFU group [35].

In a prospective study, Jones et al. looked at 100 patients with whole-gland S-HIFU and initially reported a negative 12-month biopsy rate of 81% [25]. However, during post-trial follow-up, they noted the mean PSA at 2 years was 1.1 ng/mL in 33 patients, which suggested uncertainty regarding long-term oncologic outcomes. In Hostiou et al., we saw a unique comparison of outcomes between patients treated with whole-gland ablation and those treated with hemi-ablation focal therapy [23]. At 6 years, the treatment failure-free survival rate was 41%, PFS was 45%, OS was 93%, CSS was 98%, and MFS was 80%. The PFS rate did not significantly differ between patients who underwent whole-gland treatment and those treated with focal S-HIFU (*p* = 0.604).

Kanthabalan et al. introduced a new parameter to assess survival outcomes when studying focal S-HIFU, known as the composite endpoint, which includes the occurrence of BCR, positive imaging and biopsies, starting systemic therapy, the presence of metastases, and prostate cancer-related death [28]. This new parameter aimed to more accurately capture treatment failure in the early to medium-term after focal therapy. The authors believed the Phoenix criteria was not validated in the focal salvage context and was prone to influence by ADT use prior to focal salvage therapy. This was stated to be the case for a substantial number of patients in their sample, necessitating an adjustment. BCR-free survival at 3 years was found to be 48% for the patients who underwent focal salvage HIFU. When stratified by D’Amico risk groups for pre-salvage, the estimates of BCR-free survival at 3 years were 100% for low, 61% for intermediate, and 32% for the high-risk groups. For patients who were considered PSA responders (defined as achieving a PSA nadir of ≤0.5 ng/mL), BCR occurred in 12% (18/150), and the estimated BCR-free survival after three years was about 80%. As seen in prior studies, a PSA nadir of below 0.5 ng/mL was associated with increased disease-free survival. Additionally, the BCR-free survival at 2 years for patients who had redo HIFU was 66%.

The only study to compare S-HIFU and S-RP was conducted by Devos et al., which compared two groups of 27 and 25 patients who underwent the above-mentioned treatments, respectively [24]. They showed no significant differences in estimated 5-year OS (80.9% versus 61.9%, *p* = 0.24), 5-year CSS (84.0% versus 74.0%, *p* = 0.36), and 5-year MFS (60.3% versus 55.2%, *p* = 0.55) for S-HIFU versus S-RP. However, they did note that S-RP patients were younger and healthier, which could have skewed the overall analysis. More recent data by Dason et al. showed a recurrence-free survival (RFS) rate of 66.3% at 2 years and 51.6% at 5 years after S-HIFU [26]. In addition, they examined six pre-specified predictors and found that only one, an undetectable PSA nadir, was a significant predictor of improved RFS (*p* < 0.001).

In summary, current studies on S-HIFU following primary radiotherapy have shown that the 5-year RFS averages are around 50%, and a PSA nadir of less than 0.5 ng/mL is correlated with RFS. The results of these studies signify the potential role of S-HIFU after radio-recurrent PCa as an alternative to S-RP, demonstrating acceptable disease control. Additional high-quality evidence from randomized clinical trials for select salvage patients across different treatment modalities is required in order to definitively understand outcomes from S-HIFU.

### 3.3. Urinary Functional Outcomes

Post-surgical incontinence is a major challenge associated with salvage procedures following radio-recurrent PCa, as evident post-RP [7]. With improved continence outcomes, S-HIFU may demonstrate increased quality of life with adequate disease control. A summary of functional outcomes following S-HIFU is presented in Table 3; however, given the heterogeneity of continence evaluation and reporting methods, the results should be interpreted with caution and within the context of each study. Kanthabalan et al. demonstrated great continence following whole-gland S-HIFU, especially in those who were pad-free at baseline, with 88% (42/48) remaining pad-free at 2 years [28]. Additionally, of those who were drip-free urinary continent at baseline (34/48), 67.6% (23/34) remained drip-free at 2 years postoperatively. At 12 months following S-HIFU treatment, Jones et al. reported that nearly 50% of patients experienced considerably lower urinary tract symptoms [25]. Functional outcomes in terms of urinary incontinence were reported as mild in eighteen men, moderate in twenty-five, and severe in four. Additionally, a total of 21 patients required pads at 12 months following treatment.

Crouzet et al. reported more favorable results, with 57.7% of patients being pad-free [20]. They identified 21% of patients who required one pad/day, 12% of patients who required two pads/day, and 9% of patients who required three or more pads/day. With the specific post-RT parameters introduced in 2002, many long-term complications were decreased, including a decrease in grade II or grade III incontinence from 32% to 19%. Focal S-HIFU showed lower rates of severe incontinence in four out of forty-eight (8%) patients, eight (17%) patients required one pad a day, and thirty-six (75%) were pad-free [31]. Song et al. demonstrated outstanding results during their follow-up period after whole-gland S-HIFU, with only three patients experiencing grade I incontinence and one with grade II; however, the reported incontinence was resolved after pelvic floor muscle exercises [32]. In another study on patients who underwent whole-gland S-HIFU, Siddiqui et al. did not report specific continence data, but they did report a significant increase in the IPSS scores [30]. In a long-term follow-up study, Shah et al. reported that only a third of patients had any pad use within an average of 80 months following S-HIFU [29].

In a comparative study, Devos et al. reported the functional outcomes of S-HIFU versus S-RP [24]. They observed a notable difference in pad-reliance after one year, favoring S-HIFU (22% versus 56%, *p* = 0.01). In a comparison of whole-gland versus focal S-HIFU, incontinence (irrespective of grade), bladder outlet obstruction (BOO), and complications of grade ≥ III were decreased in the hemi-ablation group compared to the whole-gland ablation (13% versus 54%; 13% versus 46%; and 13% versus 63%, for each category, respectively; all *p*-values were significant). In this study, before S-HIFU, 47 patients (94%) were continent, but after S-HIFU, only 29 patients (58%) remained continent. Patients with grade I incontinence increased from one (2%) before S-HIFU to four (8%) after S-HIFU. Grade II incontinence was seen in three patients (6%) post-S-HIFU but none pre-HIFU. Grade III incontinence increased from two patients (4%) before the procedure to fourteen patients (28%) afterward. Overall, when viewing continent to grade I incontinence as a single group, this group decreased from forty-eight (96%) before S-HIFU to thirty-three (66%), with the patients transitioning to the grade II to III incontinence group, which saw an increase from two (4%) before S-HIFU to seventeen (34%) after S-HIFU. The study also noted that over half of their patients (11/18) with BOO also developed grade II/III incontinence, indicating a correlation between BOO and severe incontinence (*p* = 0.02).

The location of the recurrence should be considered when choosing the salvage treatment approach; HIFU may be preferred, particularly for posterior lesions, due to its localized, conservative approach that aims to maximize functional outcomes and minimize tissue damage [21]. In summary, salvage procedures for radio-recurrent PCa are generally associated with compromised urinary continence outcomes. Nevertheless, S-HIFU presents more favorable outcomes and better quality of life compared to other treatment modalities, such as S-RP. It is worth mentioning that when comparing outcomes among S-HIFU and S-RP or other salvage focal therapy modalities, the results should be interpreted with caution, given the significant heterogeneity in existing reports when it comes to patient selection, timing, and clinical characterization, with most studies focusing on only one treatment modality without the robust framework of a randomized controlled trial.

### 3.4. Sexual Function Outcomes

Reporting of sexual function following S-HIFU is inconsistent between studies, with many forgoing it outright. Reports of sexual function following S-HIFU vary inconsistently across different studies. An overview of the reports on sexual function can be seen in Table 3, with studies predominantly using the IIEF questionnaire to assess potency. The majority of patients expectedly experienced a degree of sexual dysfunction following their primary treatment, which further contributed to the heterogeneity of baseline sexual function prior to salvage therapy, making it difficult to ascertain the degree of decline in potency attributable to S-HIFU. In a study of 100 participants, Jones et al. reported that out of patients who reported erectile function (*n* = 47) after primary treatment, only 12 (26%) maintained their potency following S-HIFU [25]. Kanthabalan et al. stated that about 12/31 of the men in their study initially reported having adequate erections for penetration, while this rate changed to 7/12 after S-HIFU treatment. These results highlight the challenges associated with interpreting functional data, given the missing information and the reliability of questionnaires [28]. In another recent study, the rate of erectile dysfunction rose from 50% to 76% following S-HIFU. In this study, about half of the patients who initially had IIEF-5 scores of ≥17 managed to maintain these scores at the 12-month follow-up [23]. Other studies report a comparable decrease in IIEF score following treatment, as expected [26,33].

### 3.5. Perioperative and Long-Term Complications

One of the key advantages of focal therapy compared to radical treatments is minimizing morbidity and improving perioperative outcomes. However, these procedures are not devoid of complications, especially in the post-radiotherapy setting. Baco et al. reported few complications, possibly due to their small sample size (*n* = 48) and focal-gland approach [31]. Two patients were reported to have delayed pubic bone osteitis, with one of the cases developing a pubovesical fistula 23 months after hemi S-HIFU. Shah et al. demonstrated a relatively high rate of bladder outlet obstruction, with more than half of their 50 patient samples requiring either a transurethral resection or urethral dilatation [29]. Data on repeat S-HIFU were also described in this study, with two out of forty-one developing a recto-urethral fistula after a single HIFU and one out of nine experiencing a fistula in patients requiring redo-salvage HIFU. Additionally, three patients experienced osteonecrosis of the pubic symphysis. In a prospective study conducted on 81 men, Siddiqui et al. reported a low rate of 3.7% rectal fistulization, but reported a high overall rate of complications, with 223 complications occurring within six months after surgery [30]. However, many of these were relatively low-grade, with one hundred and ninety-five being Clavien grade I, compared to seven grade III and one grade IVa complications.

In a prospective study, Dason et al. reported the functional outcomes of S-HIFU in a small cohort of 24 patients. In their work, they observed a single complication—one patient developed a urethral stricture nine months after receiving salvage HIFU treatment. This finding suggests a possible extended period for stricture development post-HIFU surgery [26]. Hostiou et al. showed similar outcomes in their 50-patient retrospective study, with one case of pubic osteitis and two cases of recto-urethral fistulae [23]. Interestingly, functional outcomes were often impacted by post-BT versus post-EBRT parameters. Of the eight patients who experienced BOO post-BT, seven experienced repeat BOO after whole-gland S-HIFU. The incidence of grade II/III incontinence was notably lower when post-BT parameters were used versus post-EBRT, with a reduction from 62% to 34% (*p* = 0.015). In the focal gland subgroup, there were no reported cases of de novo BOO, reinforcing the potentially lower rate of complications from focal therapy. A significant difference was seen in Clavien-Dindo grade III or higher complications with 7/27 S-HIFU patients versus 12/25 S-RP [24]. Of note, a higher number of BOO was seen with S-HIFU versus an increased rate of recto-urinary fistulas being seen in the S-RP group.

Crouzet et al. fielded the largest study in this setting, with over 400 patients ranging from 1995 to 2009 [20]. In this sample population, they demonstrated a drastic change in complication rates with the adoption of specific post-RT parameters for HIFU (given the decreased vascularity of previously radio-treated tissue), showing a two-thirds decrease in artificial urinary sphincter implantation, BOO rates halved to 15%, and a dramatic decrease in recto-urethral fistulas from 9% to 0.6%. The authors did mention that a lack of a control group limited the study due to its retrospective nature. In a 2017 retrospective study involving 150 patients, Kanthabalan et al. reported that approximately 11% experienced urinary infections and 8% had bladder neck strictures. Additionally, only three patients developed a recto-urethral fistula, and just one experienced osteitis pubis following a single HIFU procedure [28]. In their multi-center retrospective study of 100 men, Jones et al. gives a comprehensive look at adverse-events, using Common Terminology Criteria for Adverse Events (CTCAE) classifications to grade complications [25]. Half of the patients experienced UTIs and urinary retention, with the majority being grade II in severity. Over a third of the patients experienced mild hematuria, and cases of severe BOO were relatively low at 3%, for a total of 17%.

### 3.6. Specific Indications for Select Patients Based on Location of Reccurence or Post-Radiation Clinical Parameters

In recent years, there has been increasing evidence of the favorable functional and oncological outcomes for hemi-ablation sHIFU in select patients with unilaterally recurrent PCa, confirmed by multiparametric MRI and targeted biopsies [23,38]. In a prospective study by Aoun et al., hemi-ablation HIFU proved to be an effective alternative to whole-gland treatment in select patients with unilateral radio-recurrent PCa, with a median follow-up time of 41.5 months. The BCR-free survival rates at 24 and 36 months were 75% and 60%, respectively, in addition to providing limited urinary and rectal morbidity, thus, favorable in terms of functional outcomes as incontinence occurred in 2/10 patients while the remaining patients were pad-free [38]. Hostiu et al. also reported that hemi-ablation reduced incontinence, recto-urethral fistulas, and Clavien-Dindo ≥ III complications, thereby highlighting the utility of hemi-ablation for cases in which there is early detection of unilateral recurrence [23].

Regarding pre-S-HIFU clinical parameters, Siddiqui et al. reported that a Gleason score (6/7 vs. 8 or greater) nearly reached statistical significance (*p* = 0.059) as a predictor of biochemical recurrence-free survival. However, variations in pre-salvage PSA levels (less than 5 vs. 5 to 10 vs. greater than 10 ng/mL) did not have a statistically significant valence on outcomes, which could be due to the small number of patients with PSA > 10 ng/mL in the study. This trend points to the importance of tumor aggressiveness, as indicated by Gleason score, in predicting the success of S-HIFU [27].

## 4. Conclusions

In conclusion, there is a lack of consensus on the optimal treatment modality for radio-recurrent prostate cancer patients, partly due to the lack of robust data and heterogeneity of studies. Nonetheless, salvage HIFU emerges as an effective treatment strategy in this setting, with lower morbidity rates compared to radical treatments. Additional randomized clinical trials are needed, focusing on select salvage patients across different treatment modalities.

## Figures and Tables

**Table 1:** Overview of salvage HIFU studies included in this review.

Author and Year of Publication	Study Type	Country	Focal/Whole Gland	Sample Size	Median Age	Primary Radiation Treatment Type	Mean Radiation Dose (SD), Cycle, or Seed Type	Androgen Deprivation Therapy	Time from Primary Treatment to Salvage
Nair et al., 2021 [35]	Retrospective	Canada	Whole	113	63.3	BT and EBRT	NA	20.4%	14.3 years
Hostiou et al., 2019 [23]	Retrospective	France	Whole (hemi-ablation when unilateral in some cases)	50	62.1	BT	Iodine-125 (96%) Iridium-192 (2%) Unknown (2%)	16%	NR
Devos et al., 2019 [24]	Retrospective	Belgium	Whole	27	69.9	EBRT	EBRT: 70.7 ± 2.7 BT: 70.1 ± 3.3	31%N-AD	NR
Jones et al., 2018 [25]	Prospective	USA	Whole	100	70	EBRT	NA	None within 3 months of HIFU	24 mo minimum
Dason et al., 2018 [26]	Prospective	Canada	Whole	24	68	88%: EBRT 12%: BT	NA	25%	NR
Siddiqui et al., 2017 [27]	Prospective	UK	Whole	81	69	82.7%: EBRT 16.1%: BT	NA	26.9%	NR
Kanthabalan et al., 2017 [28]	Retrospective	UK	Focal	150	69.8	96.7%: EBRT 3.3%: EBRT + BT	64 in 32 fractions	45.3%: pre-salvage	80 mo
Crouzet et al. 2017 [20]	Retrospective	France	Whole	418	68.6	EBRT	NA	None within 3 months of HIFU	5.1 ± 2.7 yrs
Shah et al., 2016 [29]	Prospective	UK	Whole	50	68	EBRT	Range: 50–72 in 20–35 fractions, Median 57.5	52% (after biochemical failure)	80 mo
Siddiqui et al., 2015 [30]	Retrospective	UK	Whole	65	71	93.8%: EBRT 6.2%: BT	NA	21%	NR
Yutkin et al., 2014 [33]	Prospective	Canada	Whole	19	60	BT	Iodine-125 (94.7%) high-dose rate Iridium (5.3%)	27% N-AD	72
Baco et al., 2014 [31]	Prospective	France and Norway	Focal	48	68.8	95.8%: EBRT 4.2%: BT	72.5 ± 3.3	22.9%: N-AD	NR
Song et al., 2014 [32]	Retrospective	Korea	Whole	13	68	EBRT	NA	8/13 pre-salvage for 2 months	32.7 months median
Rouvière et al., 2013 [34]	Retrospective	France	Whole	46	NR	EBRT	NA	N-AD32%	NR

EBRT: external beam radiation therapy, NR: not reported, BT: brachytherapy, HIFU: high intensity focused ultrasound.

**Table 2:** Overview of studies reporting oncologic outcomes on salvage HIFU following radiation therapy.

Author and Year of Publication	Oncologic Outcomes	Key Findings
Nair et al., 2021 [35]	Median OS S-HIFU 17.4 yr vs. 12.8 yr for NST (no salvage treatment) although not statistically significant	52 deaths (31 from PCa) for NST (no salvage therapy) group vs. 18 deaths (9 from PCa) for S-HIFU; however, no significant difference in CSS or OS likely due to reduced sample size and shorter follow-up of S-HIFU group.
Hostiou et al., 2019 [23]	6-yr FFS: 41% 6-yr PFS: 45% 6-yr OS: 93% 6-yr CSS: 98% 6-yr MFS: 80%	There was no difference between hemi-ablation and whole-gland treatment in terms of PFS.
Dason et al., 2018 [26]	5-yr RFS 51.6%	RFS 2 yr: 66.3%. No difference was seen in RFS between patients initially treated with ERBT versus brachytherapy.
Siddiqui et al., 2017 [27]	5-yr OS of 88%. 5-yr CSS of 94.4%.	At 53.5 ± 31.6 months follow up, the median BRFS was 63 months. At 6 months, 22/63 men had residual disease.
Shah et al., 2016 [29]	5-yr PFS of 31%. 5-yr OS of 87%.	Removal of PSA non-responders resulted in a 5-year PFS of 37%. Postoperative PSA nadir was significantly associated with PFS and OS.
Kanthabalan et al., 2017 [28]	3-yr BDFS of 48%	In PSA responders, achieving a PSA nadir of ≤0.5 ng/mL correlated with a BF rate of 12% (18/150). Redo HIFU subgroup had a 2-year BDFS of 66%. Whole gland pad free at BDFS 36 months was 78%.
Jones et al., 2018 [25]	NA	12-month biopsy was negative in 63 (81%) men. Mean PSA at 2 years was 1.1 ng/mL in 33 patients.
Devos et al., 2019 [24]	5-yr OS 80.9% 5-yr CSS 84% 5-yr MFS 60.3%	No significant differences found in S-HIFU versus S-RP for 5-year OS, CSS, and MFS.
Crouzet et al. 2017 [20]	7-yr OS 72%, 7-yr CSS 82%, 7-yr MFS 81%, 5-yrBRFS 49%	S-HIFU 7-year CSS and MFS rates of >80% with significant morbidity. Concluded that S-HIFU should be initiated early following EBRT failure
Yutkin et al., 2014 [33]	4-yr BRFS 73.6%	No significant association was found between biochemical failure, pre-HIFU Gleason score, PSA nadir, or other variables.
Baco et al., 2014 [31]	PFS rates at 12 mo: 83%, 18 mo: 64%, and 24 mo: 52%	The D’Amico risk group before EBRT did not correlate with 18-month PFS. 18-month PFS rate was significantly associated with Gleason score during post-EBRT recurrence (≤7, 82%; ≥8, 34%; *p* = 0.047), and by the PSA level before HSH (≤4 ng/mL, 80%; >4 ng/mL, 49%, *p* = 0.002).
Song et al., 2014 [32]	5-yr BRFS 53.8%	BCR after salvage HIFU correlated with higher pre-EBRT PSA, pre-HIFU PSA, and short time to nadir in univariate analysis only.
Rouvière et al., 2013 [34]	PFS was 42% at 2-yr and 31% at 4-yr	PSA level at HIFU treatment and tumor extension anterior to the urethra, as assessed by MRI, were independent predictors of salvage HIFU failure.

BRFS: biochemical recurrence free survival, RFS: recurrence free survival, PFS: progression free survival, OS: overall survival, EBRT: external beam radiation therapy, PSA: prostate-specific antigen, HIFU: high intensity focused ultrasound, BCR: biochemical recurrence, CSS: cancer-specific survival.

**Table 3:** Overview of studies reporting functional outcomes on salvage HIFU following radiation therapy.

Author and Year of Publication	Incontinence (General)	Sexual Function	Key Findings
Devos et al., 2019 [24]	22.2% at 12 mo	NR	Complications were significantly lower in S-HIFU than S-RP, with S-HIFU patients more commonly experiencing urinary retention. Pad-dependent status differed at 22.2% for S-HIFU versus 56.0% for S-RP (*p* = 0.0104).
Crouzet et al. 2017 [20]	Grade > II Initially 32%; down to 19%.	NR	With post-RT specific HIFU parameters, complication rates improved over the course of the study. Incontinence (Grade II/III) decreased from 32% to 19%; recto-urethral fistula changed from 9% to 0.6%; BOO or stenosis decreased from 30% to 15%.
Jones et al., 2018 [25]	47%	12/47 maintained erectile function post S-HIFU.	Treatment related Grade III adverse events developed early in the trial and appeared related to operator experience. No life-threatening treatment-related deaths or adverse events reported.
Kanthabalan et al., 2017 [28]	22%	Baseline of 12/31 capable of penetration. Following treatment, 7/12 reported erection capable of penetration.	Of those who were pad-free at baseline, (42/48) remained pad-free at 2 years. UTI (11.3%), bladder neck stricture (8%), and recto-urethral fistula (8%) were the most common complications. 12/31 (38%) of patients initially reported having adequate erections for penetration, while this rate changed to 7/12 (58%) after S-HIFU.
Siddiqui et al., 2017 [27]	IPSS significantly increased from baseline at 45 days (*p* < 0.001).	IIEF-5 scores significantly decreased from baseline.	No significant changes were observed. IPSS scores increased, IIEF-5 scores were reduced, while SF-36 score did not change significantly post S-HIFU.
Siddiqui et al., 2015 [30]	7.5%	NR	Pre-HIFU IIEF score among patients was 8.6 (±: 7.9), which disqualified them from receiving nerve-sparing treatment. Post-HIFU IIEF scores were 3.4 (±4), 5.1 (±5), and 5.4 (±6.5) at 45, 90, and 180 days, respectively.
Shah et al., 2016 [29]	31%	Non-significant decrease in IIEF-15 score after treatment.	Side effects with this study were equal to other salvage therapies in high-risk groups.
Baco et al., 2014 [31]	25%	IIEF-5 score significantly decreased from 11.2 (SD: 8.0) to 7.0 (SD: 5.8).	Average IPSS scores increased and average IIEF scores decreased after S-HIFU. ICS A and B scores increased.
Song et al., 2014 [32]	30.8%	NR	4 patients reported incontinence after treatment. No observed incidence of acute urinary retention, urinary tract infection, anal incontinence, rectal injury, urethro-rectal fistula, or urethral stricture.
Yutikin et al., 2014 [33]	31.6%	IIEF-5 scores above 20 decreased from 4 pre-treatment to 2 at 6 month follow up.	Administration of ADT before HIFU and longer interval between BT and S-HIFU were both associated with higher complication rates. UTI was the most common complication.
Hostiou et al., 2019 [23]	Grade I: 20/35 whole gland, 13/15 focal. Grade II-III: 15/35 whole gland, 2/15 focal.	ED rate increased from 50% to 76% from pre- to post-op. Almost half of the 25 patients with IIEF-5 scores of ≥17, were maintained at 12 months.	Incontinence, BOO and Grade ≥ III complications decreased significantly in hemi-ablation versus whole-gland intervention.
Dason et al., 2018 [26]	NR	Median IIEF-15 score decreased from 43 pre-treatment to 19 post-treatments.	Only one complication was observed. Median IPSS increased from a baseline of 8 to 24 at a median year 1 average and 17 at a median 2-year average.

IPSS: International Prostate Symptom Score, NR: not reported, BT: brachytherapy, IIEF: International Index of Erectile Function, BOO: bladder outlet obstruction.

## Data Availability

Not applicable.
